# Diagnosis and prevalence of two new species of haplosporidians infecting shore crabs *Carcinus maenas*: *Haplosporidium carcini* n. sp., and *H. cranc* n. sp.

**DOI:** 10.1017/S0031182020000980

**Published:** 2020-09

**Authors:** Charlotte E. Davies, David Bass, Georgia M. Ward, Frederico M. Batista, Sophie H. Malkin, Jessica E. Thomas, Kelly Bateman, Stephen W. Feist, Christopher J. Coates, Andrew F. Rowley

**Affiliations:** 1 Department of Biosciences, College of Science, Swansea University, Swansea SA2 8PP, Wales, UK; 2 Department of Life Sciences, The Natural History Museum, Cromwell Road, London SW7 5BD, UK; 3 Animal and Human Health, Centre for Environment, Fisheries and Aquaculture Science (CEFAS), Barrack Road, The Nothe, Weymouth DT4 8UB, UK

**Keywords:** Ascetosporea, *Carcinus maenas*, disease connectivity, Haplosporida, *Haplosporidium carcini*, *Haplosporidium cranc*, new species, parasite, phylogeny

## Abstract

This study provides a morphological and phylogenetic characterization of two novel species of the order Haplosporida (*Haplosporidium carcini* n. sp., and *H. cranc* n. sp.) infecting the common shore crab *Carcinus maenas* collected at one location in Swansea Bay, South Wales, UK. Both parasites were observed in the haemolymph, gills and hepatopancreas. The prevalence of clinical infections (i.e. parasites seen directly in fresh haemolymph preparations) was low, at ~1%, whereas subclinical levels, detected by polymerase chain reaction, were slightly higher at ~2%. Although no spores were found in any of the infected crabs examined histologically (*n* = 334), the morphology of monokaryotic and dikaryotic unicellular stages of the parasites enabled differentiation between the two new species. Phylogenetic analyses of the new species based on the small subunit (SSU) rDNA gene placed *H. cranc* in a clade of otherwise uncharacterized environmental sequences from marine samples, and *H. carcini* in a clade with other crustacean-associated lineages.

## Introduction

Haplosporidians (Rhizaria, Retaria, Ascetosporea, Haplosporida) are most often regarded as parasites of molluscs, the best known including *Bonamia* spp. in the flat oyster *Ostrea edulis* (Abollo *et al*., 2008), *Haplosporidium nelsoni* (MSX disease) and *Haplosporidium costale* (SSO disease) in both *Crassostrea virginica* and *Crassostrea gigas* (Burreson and Ford, 2004; Wang *et al*., 2010), *Minchinia* spp. in various molluscs (Ramilo *et al*., 2017), and the hyperparasitic *Urosporidium*, which infects platyhelminth parasites of molluscs (Carballal *et al*., 2005), as well as free-living annelids (Caullery and Mesnil, 1905). Other species are known from a range of molluscan hosts, some causing disease and mortalities, others apparently persisting at low levels eliciting few clinical signs in their hosts – perhaps representing spill-over (Arzul and Carnegie, 2015; Ramilo *et al*., 2017; Ward *et al*., 2019).

More recently, *Haplosporidium* spp. have been identified as parasites of crustaceans, initially with the characterization of *Haplosporidium littoralis* in the shore crab *Carcinus maenas* (Stentiford *et al*., 2013), and subsequently *H. diporeae*, *H. echinogammari* and *H. orchestiae* in amphipods (Winters and Faisal, 2014; Urrutia *et al*., 2019). Environmental sequencing (eDNA) studies show that many other haplosporidian lineages exist, associated with molluscan and crustacean (putative) hosts, and in the surrounding water, sediment and soil (Hartikainen *et al*., 2014; Ward *et al*., 2018, 2019; Urrutia *et al*., 2019). However, crustaceans are generally overlooked as potential hosts of haplosporidians and ascetosporeans more widely (Bass *et al*., 2019). A wider range of hosts is now being investigated using both eDNA and host survey approaches to better understand haplosporidian diversity, distribution, life cycles, contribution to ecological processes and disease connectivity. Because of these efforts, a much greater diversity of haplosporidian sequences has been revealed. Although most of the eDNA-generated sequences cannot be associated with specific hosts, the large number of sequences from disparate sample types allows the inference of ecological patterns such as habitat preference, temporal variation, possible alternative hosts and lifecycle stages. Additionally, the proliferation of genetic data (so far mostly 18S rRNA gene regions) provides much-needed material for phylogenetic reconstructions, which help to place characterized taxa into a more comprehensive evolutionary framework.

Integrating data from these different sources suggest that morphological variation within Haplosporida is not a very reliable indicator of phylogenetic affinities (Burreson and Reece, 2006). On the other hand, it should be clearly acknowledged that the 18S gene alone is insufficient to resolve many nodes on the haplosporidian phylogeny. As is the case for most other protistan groups, a robust phylogeny will likely only be possible based on more comprehensive taxonomic sampling approaches and a larger number of genes. Species definitions do not require unambiguous morphological differences between taxa, which is particularly challenging for protistan parasites as the full range of life stages cannot be accessed for study; free-living or dispersal stages are extremely elusive, and reproductive stages such as sporulation may not be present in all hosts in the lifecycle.

In this study, we describe two novel species of Haplosporida. Both species were found infecting shore crabs *C. maenas* collected from Mumbles Pier in Swansea Bay (South Wales, UK). We provide in-depth histological and phylogenetic information for novel species description.

## Materials and methods

### Survey site and sample collection

Shore crabs, *Carcinus maenas,* were sampled from the South Wales coast, UK at two distinct locations, the Prince of Wales Dock, Swansea (51°37′8.76″N, 3°55′36.84″W) and Mumbles Pier (51°34′8.958″N, 3°58′33.297″W) according to Davies *et al*. (2019). For 12 months from November 2017 to October 2018, the shore crab population was surveyed at both locations. Briefly, strings of baited traps were deployed and immersed for 24 h, retrieved and 50 crabs were chosen randomly, bagged individually and transported back to the laboratory on ice. All crabs were examined using haemolymph preparations for primary screening using the phase contrast optics of an Olympus BX41 microscope. If positive, polymerase chain reaction (PCR) and histology were then used as confirmatory measures. In addition to these clinical positives, a sub-sample of 324 animals were screened using PCR to account for any sub-clinical infections.

### DNA extraction and quantification

Genomic crab DNA was extracted from 100 *μ*L of thawed haemolymph using Qiagen Blood and Tissue Kits (Qiagen, Hilden, Germany) following the manufacturer's instructions. Extracted DNA was quantified using a Qubit^®^ dsDNA High Sensitivity Assay Kit and Qubit^®^ Fluorometer (Invitrogen, California, USA).

### PCR and sequencing conditions

Initial haplosporidian-specific PCR reactions were carried out using a nested protocol modified from Hartikainen *et al*. (2014), producing amplicons of ~670 bp. The first-round PCR (10 *μ*L total reaction volume) used primers C5fHap and Sb1n and 1 *μ*L template DNA (*ca*. 50–200 ng/*μ*L) followed by a second round (25 *μ*L total reaction volume) using primers V5fHapl and Sb2nHap and 2.5 *μ*L amplicon DNA from first-round PCR. Both rounds of PCR used 2X Master Mix (New England Biolabs), oligonucleotide primers synthesized by Eurofins (Ebersberg, Germany), and were performed on a BioRad T100 PCR thermal cycler (primers and cycling conditions shown in Table 1). Products derived from the second round PCR were visualized on a 2% agarose/TBE gel with GreenSafe premium nucleic acid stain (NZYTech, Portugal). Sanger sequencing was performed using both forward and reverse primers synthesized by Source BioScience (Nottingham, UK) and Eurofins. Sequences were deposited in GenBank under accession numbers MN846346-MN8463561.

**Table 1.** Forward and reverse primer sequences used for the amplification of Haplosporidia by PCR. Each PCR run included initial denaturation and final extension steps, according to the first and final temperatures, respectively, noted in the thermocycler settings.

Pathogen	Primers				Thermocycler settings			References
	Dir.	Name	Sequence (5′–3′)	Final conc. (*μ*m)	Temp (°C)	Time	No. of cycles	
*Haplosporidia* sp.	Fwd	C5fHap	GTAGTCCCARCYATAAACBATGTC	1	95	5 min	30	Hartikainen *et al*. (2014)
(round 1)	Rev	Sb1n	GATCCHTCYGCAGGTTCACCTACG	1	95	30 s		
					65	45 s		
					72	1 min		
					72	10 min		
*Haplosporidia* sp.	Fwd	V5fHapl	GGACTCRGGGGGAAGTATGCT	1	95	5 min	30	Hartikainen *et al*. (2014)
(round 2)	Rev	Sb2nHap	CCTTGTTACGACTTBTYCTTCCTC	1	95	30 s		
					65	45 s		
					72	1 min		
					72	10 min		
*Haplosporidia* sp.	Fwd	HapGenFor33	TTGYCTYAAAGATTAAGCCATGCA	0.5	95	5 min	40	Ward *et al*. (2019)
(V2 to V9	Rev	Sb2nHap	CCTTGTTACGACTTBTYCTTCCTC	0.5	95	30 s		Hartikainen *et al*. (2014)
variable regions					58	1 min		
of SSU)					72	90 s		
					72	12 min		

Each novel haplosporidian sequence type was extended to include the V2–V9 variable regions of the small subunit using primers HapGenFor33 (Ward *et al*., 2019) and Sb2nHap (Hartikainen *et al*., 2014). Reactions were performed in 50*μ*L volumes comprising 1X Promega colourless buffer, 2.5 mm MgCl_2,_ 0.4 mm dNTPs, 0.2 mg bovine serum albumin, 1.5 U GoTaq G2 (Promega, USA) and 2.5 *μ*L template DNA. Resultant amplicons (ca. 1700 bp) were visualized on a 1% agarose-TAE gel stained with GelRed (Biotium, USA). and bidirectionally Sanger sequenced using the primers used for amplification. Sequences were deposited in GenBank under accession numbers MT311214 – MT311215.

### Phylogenetic analyses

The two new near full-length small subunit sequences were blastn-searched against GenBank and the top 10 best matches downloaded. These 12 sequences were added to a phylogenetically comprehensive haplosporidian alignment using the L-ins-i algorithm in MAFFT (Katoh *et al*., 2017). The dataset was refined on the basis of this tree, resulting in an alignment of 50 sequences. Maximum Likelihood phylogenetic analyses were carried out using RAxML BlackBox v.8 (Stamatakis, 2014) (GTR model with CAT approximation; all parameters estimated from the data). A Bayesian consensus tree was constructed using MrBayes v.3.2.5 (Ronquist *et al*., 2012). Two separate MC3 runs with randomly generated starting trees were carried out for 4 million generations each with 1 cold and 3 heated chains. The evolutionary model applied a GTR substitution matrix, a 4 category autocorrelated gamma correction and the covarion model. All parameters were estimated from the data. The trees were sampled every 1000 generations and the first 1 million generations discarded as burn-in. All phylogenetic analyses were carried out on the Cipres server (Miller *et al*., 2010).

### Histology

Tissue histology was used as the secondary tool after PCR, to estimate the severity of, and potential host immune responses to, any haplosporidian infection (e.g. melanisation reactions, haemocyte encapsulation). Three pairs of gills and three portions (*ca.*0.5 cm^3^) of the hepatopancreas/gonad were excised and fixed in Davidson's seawater fixative for 24 h prior to their storage in 70% ethanol. Samples were dehydrated in a graded series of ethanol, transferred to Histoclear/Histochoice (Sigma-Aldrich, Dorset, UK) and infiltrated with molten wax using a Shandon™ automated tissue processor (Thermo Fisher Scientific, Altrincham, UK) prior to embedding. Blocks were cut at 5–7 *μ*m thickness using an RM2245 microtome (Leica, Wetzlar, Germany). Sections were mounted on glass slides using glycerine albumin and stained with Cole's haematoxylin and eosin. Stained slides were viewed and imaged using an Olympus BX41 microscope. Images were adjusted for colour balance and contrast only.

## Results

### General population observations

Overall, 1,191 crabs were sampled across the year-long survey, 603 from the Dock and 588 from the Pier. Of these crabs, 10 individuals (0.84%) were seen to contain haplosporidians *via* haemolymph preparations of live cells viewed using phase-contrast microscopy, all in the Pier location. Following this, a subsample of 324 crabs was tested for haplosporidians *via* PCR. Of these further 324 crabs analysed using PCR, 6 crabs were positive for haplosporidians at a subclinical level (1.85%), again, all at the Pier location. In total, 334 crabs were tested for haplosporidians, first using haemolymph preparations observed using phase-contrast microscopy, then PCR and histology. Crabs harbouring haplosporidians occurred broadly across the whole year, showing no seasonality, in all months except January, May, July, August and November. There was also no apparent host sex preference (9 females *vs* 7 males).

### Phylogenetic analyses

Partial 18S rRNA sequences of the crabs positive for haplosporidian by microscopical examination or group-specific PCR were aligned, showing two clearly distinct sequence types: *Haplosporidium carcini* sp. nov. and *H. cranc* sp. nov. Each crab harboured just one haplosporidian infection. These were searched using the BLASTn function against the NCBI GenBank nt database. The top match for *H. carcini* sp. nov was accession number MK070859 (87.7% identity, 95% coverage), which corresponds to a *Mytilus edulis*-infecting haplosporidian reported in Ward *et al*. (2019). The closest characterized match for *H. carcini* sp. nov was *Haplosporidium nelsoni*, accession number U19538 (85.8% identity, 99% coverage). The top match for sequence *H. cranc* sp. nov was KF208557 (87.8%), a member of novel lineage A in Hartikainen *et al.* (2014), sampled from seawater in the UK and Bulgaria. The top characterized match was *Bonamia exitiosa* (JF831802; 79.2%, 100% coverage).

**Fig. 1.**
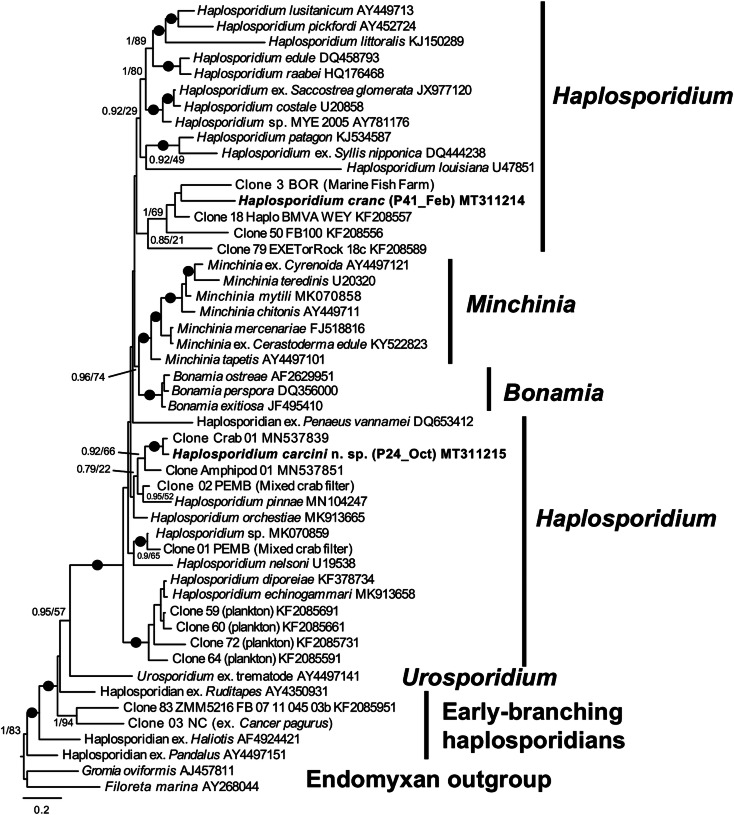
Bayesian 18S rRNA gene phylogeny showing the positions of *Haplosporidium carcini* and *H. cranc* (bold text) in relation to a broad selection of other haplosporidians, rooted on *Gromia* and *Filoreta*. Bayesian Posterior Probabilities (BPP) and Maximum Likelihood (ML) bootstrap values are indicated on nodes; black filled circles indicate values of BPP ≥0.95 and ML bootstrap ≥95%.

Bayesian and Maximum Likelihood phylogenetic analyses (Fig. 1) revealed both *H. carcini* sp. nov. and *H. cranc* sp. nov. branch within the paraphyletic assemblage of lineages that currently comprise the genus *Haplosporidium*. Both sequence types were markedly different from characterized lineages in GenBank but more closely related to eDNA sequences derived from environmental and organismal samples. *Haplosporidium carcini* was 3.2% dissimilar to a sequence type detected in shore crab samples from the Dart and Tamar estuaries (SW England), and 8% dissimilar to one from amphipods also from the Tamar estuary, all forming a clade with 0.92 Bayesian Posterior Probability support. *Haplosporidium cranc* formed a maximally supported clade in the Bayesian analysis with two other lineages amplified from filtered water samples from the Fleet Lagoon (Weymouth, England), reported in Hartikainen *et al*. (2014), and one from a finfish farm situated in an estuarine river in Borneo.

*Haplosporidium carcini* sp. nov. and *H. cranc* sp. nov. were characterised from crabs *Carcinus maenas* sampled from one location, Mumbles Pier, Swansea Bay (51°34′8.958″N, 3°58′33.297″W); South Wales, UK.

### Histopathology

**Fig. 2.**
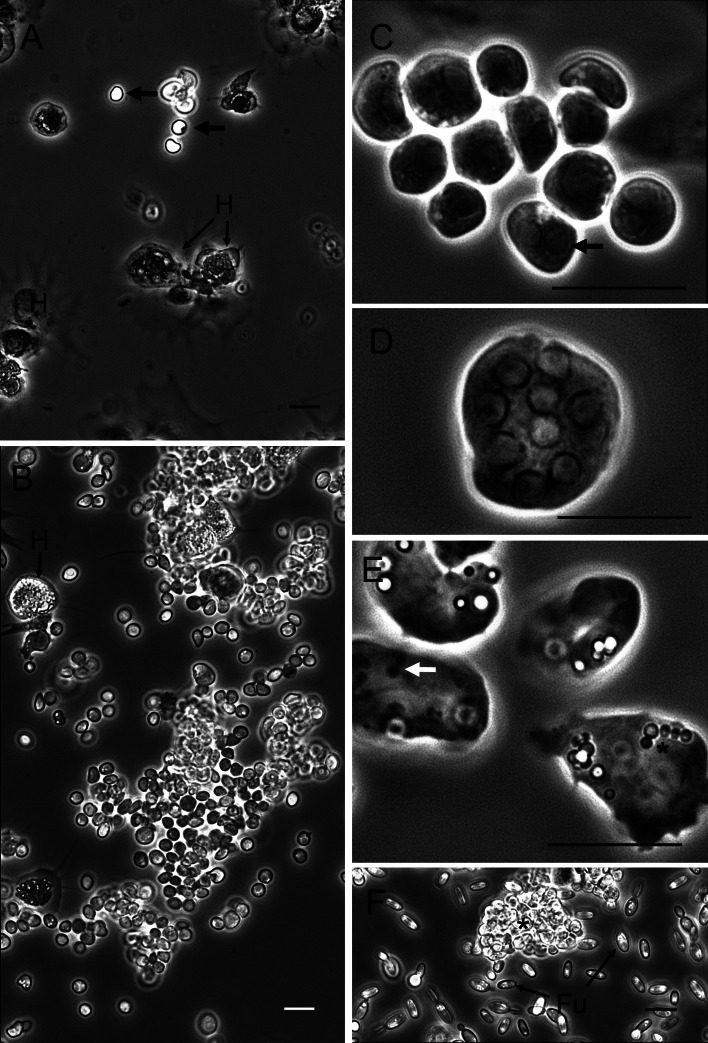
*Haplosporidium carcini*. Phase-contrast micrographs of haemolymph preparations from infected shore crabs. (A). Apparent low-grade infection with adherent haemocytes (H) and non-adherent, refractile haplosporidia (unlabelled arrows). (B). Higher severity infection with numerous uninucleate parasites and smaller number of haemocytes (H). (C). Uni- and bi-nucleate (arrow) forms of parasite. (D). Multinucleated plasmodium. (E). Spread cells of unknown origin with cytoplasmic refractile bodies (*) and bacteria-like structures (arrow). (F). Coinfection of *H. carcini* (*) and an unidentified yeast-like fungus (Fu). Scale bars = 10 *μ*m.

*Haplosporidium carcini*: The haemolymph of crabs harbouring *H. carcini* infections was cloudy-milky in consistency. Observations of fresh haemolymph preparations with phase-contrast microscopy revealed a variable number of highly phase-refractile forms of this parasite. In the case of low severity infections (as judged by the numbers of parasites in the haemolymph preparations), there were small numbers of parasites with interspersed spread haemocytes (Fig. 2A) observed, while in high severity infections the haemolymph was replete with large numbers of parasites and an apparent marked reduction in haemocytes in such preparations (Fig. 2B). The severity of infection was assessed by observing parasite numbers in haemolymph and the apparent ratio of haemocytes to parasites. Thus, it is a qualitative observation only. Similarly, histology showed more parasites in tissues of heavily infected crabs. The majority of the haplosporidians in such preparations were found to be uni- and bi-nucleate of mean diameter 4.6 *μ*m (range 3.2–5.9 *μ*m, *n* = 20; Fig. 2C). Some infected crabs had a few multinucleate (range, 3–9 nuclei/plasmodium), plasmodia 11.3 *μ*m in diameter (range 6.8–14.1 *μ*m in diameter) present in the haemolymph (Fig. 2D). The haemolymph of all infected crabs also contained highly pleomorphic cells with blunt pseudopodial extensions that were morphologically distinct to the attached and spread haemocytes (Fig. 2E). The cytoplasm enclosed small (<1 *μ*m in diameter) phase-bright rounded particles apparently within vacuoles and elongate phase-dark bacteria-like structures (Fig. 2E). Whether these cells represent altered haemocytes or a stage of this or other parasites is unclear. There was no evidence of any uptake of this parasite morphotype by host haemocytes as no intracellular forms were observed. One of the 15 crabs affected by *H. carcini* was co-infected with a yeast-like fungus (Fig. 2F), two further crabs were co-infected with the dinoflagellate parasite, *Hematodinium* (not shown) and there were 4 crabs harbouring unidentified encysted trematode parasites in the hepatopancreas.

**Fig. 3.**
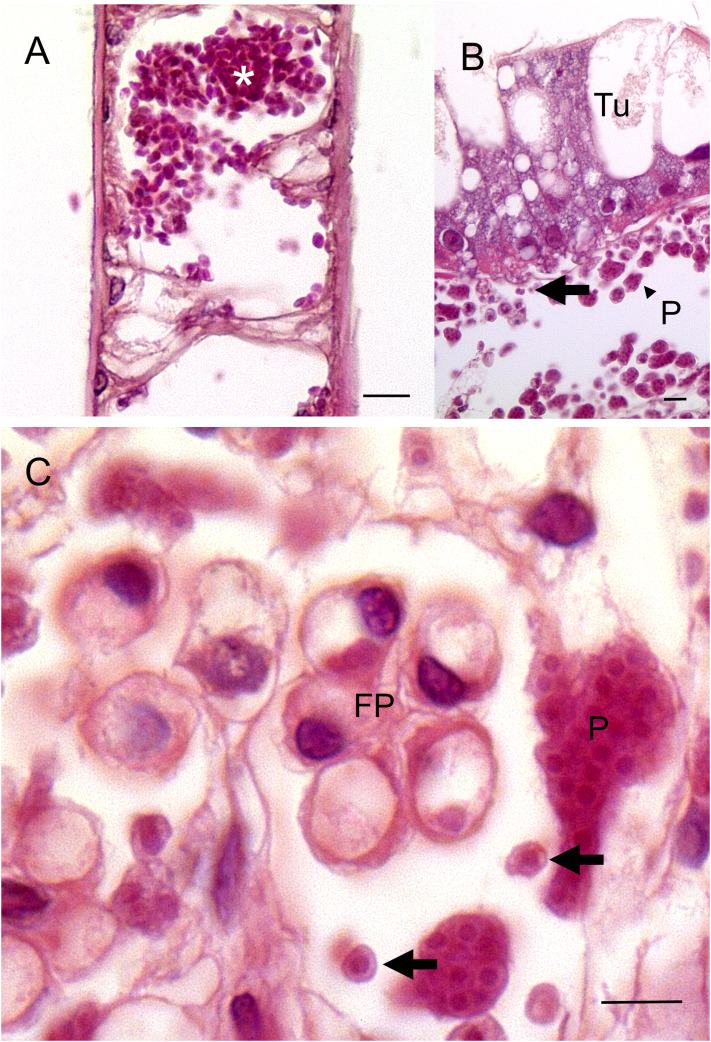
Histopathology of *Haplosporidium carcini* infections in shore crabs. (A). Section through a gill lamella of a shore crab showing numerous uninucleate parasites (*) in the haemal spaces. (B). Low power micrograph of hepatopancreas from an infected crab. Note larger plasmodia (P) and small uninucleate forms of the parasite (unlabelled arrow) in the intertubular space. Tubule (Tu). (C). High power micrograph showing the presence of multinucleate plasmodia (P) and uninuclear forms (unlabelled arrows) in the intertubular space of a hepatopancreas. Note fixed phagocytes (FP) with no clear evidence of intracellular parasites. Scale bars = 10 *μ*m.

Histological examination of crab tissues revealed the presence of both uninucleate and multinucleate plasmodia. Membrane formation around the nuclei is similar to that described in *H. littoralis*. In the case of the gills, numerous uninucleate forms were found in the haemal spaces and vessels within the central shaft and in the respiratory lamellae (Fig. 3A). There was no evidence of the uptake of such cells or breakdown products of these by the host's nephrocytes, epithelial cells underlying the cuticle or circulating haemocytes. Host response was limited to some haemocyte accumulation in haemal spaces in the hepatopancreas but with no evidence of walling-off of parasites. The interstitial spaces between the hepatopancreatic tubules were replete with uninucleate and multinucleate plasmodia (Fig. 3B and C). Both uninucleate and plasmodial stages were associated with the interstitial tissues surrounding the tubules. Uninucleate parasites were largely confined to haemal channels while plasmodia were found attached to the tubules and the fixed phagocytes within the inter-tubular space. Tubules themselves were not affected and no spore-like structures of *H. carcini* were observed in any infected tissues.

**Fig. 4.**
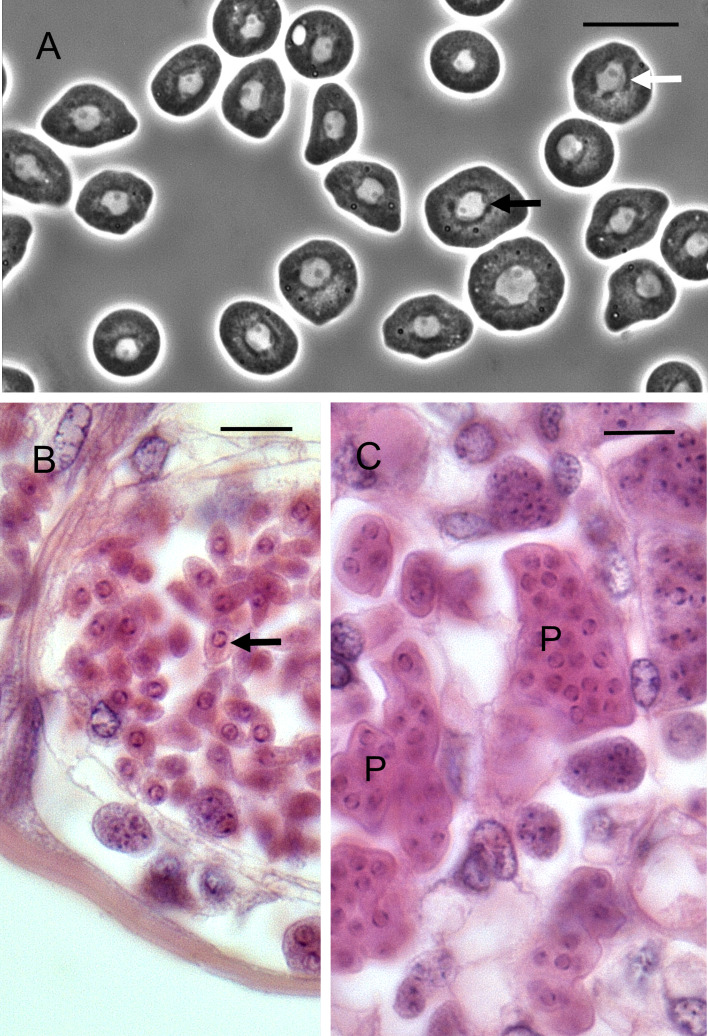
*Haplosporidium cranc* infection of a shore crab. (A). Phase-contrast micrograph showing mononucleated haplosporidians in the haemolymph of an infected crab. Note unusual chromatin arrangement (unlabelled arrows). (B). Uninucleate haplosporidians in the terminal vessel of a gill lamella. Note prominent chromatin blocks in these cells (unlabelled arrow). (C). Multinucleated plasmodial forms (P) of this parasite in the interstitial space of the hepatopancreas. Scale bars = 10 *μ*m.

*Haplosporidium cranc*: One crab (0.30%) of the 334 surveyed crabs from the Pier location had a haplosporidian infection that showed distinct morphological differences to those of *H. carcini*. The haemolymph was milky in consistency and observations of haemolymph preparations revealed numerous mono-nucleated stages of the parasite with characteristic peripheral bands of chromatin (Fig. 4A). These cells were 8.7 *μ*m in diameter (range 5.9–11.4, *n* = 20). Histological examination of the crab with this infection showed numerous mononuclear forms of the parasite in all tissues (Fig. 4B). There were also plasmodial, multinucleate forms in the interstitial spaces of the hepatopancreas (Fig. 4C). There was no clear evidence of any forms of this parasite within free or fixed phagocytes and no spore-like structures were observed.

### Taxonomic summary

*Phylum:* Retaria Cavalier-Smith (2018).

*Class:* Ascetosporea Cavalier-Smith (2002).

*Order:* Haplosporida Caullery and Mesnil (1899).

*Family*: Haplosporiidae Sprague (1979).

*Genus: Haplosporidium* Caullery and Mesnil (1899).

*Species: Haplosporidium carcini* sp. nov. Davies *et al*. (2020).

*Diagnosis:* Spherical monokaryotic and dikaryotic stages observed in haemolymph, with a mean diameter of 4.6 *μ*m (range 3.2–5.9 *μ*m). Plasmodia commonly found in connective tissue within the interstitial area of the hepatopancreas and rarely in the haemolymph mean diameter 11.3 *μ*m (range 6.8–14.1 *μ*m) with no more than 21 nuclei *per se*ction. Spore stages not observed but cannot be ruled out. Unusual pleomorphic cells were seen in haemolymph of infected crabs of unknown origin. Little host response to infection and no tissue damage apparent. No clear evidence of parasites within haemocytes or other cell types in the gills and hepatopancreas.

*Type host:* Shore crab (*Carcinus maenas*).

*Type location:* Mumbles Pier, Swansea Bay (51°34′8.958″N, 3°58′33.297″W)

*Type material:* Original slides used for this paper are stored together with biological material embedded in wax at Swansea University and the Cefas Weymouth Lab. The SSU rDNA sequence is deposited in GenBank under accession number MT311215.

*Etymology:* carcini = of the host genus *Carcinus*, in which this parasite was first detected.

*Phylum:* Retaria Cavalier-Smith (2018).

*Class:* Ascetosporea Cavalier-Smith (2002).

*Order:* Haplosporida Caullery and Mesnil (1899).

*Family*: Haplosporiidae Sprague (1979).

*Genus: Haplosporidium* Caullery and Mesnil (1899).

*Species: Haplosporidium cranc* sp. nov. Davies *et al*. (2020).

*Diagnosis:* Monokaryotic stages, 8.7 *μ*m in diameter (range 5.9–11.4 *μ*m) in haemolymph. Nuclei with peripheral bands of chromatin. Multinucleate plasmodia in gills and hepatopancreas. Spore stages not observed but cannot be ruled out. No evidence of host response to infection or tissue disruption.

*Type host:* Shore crab (*Carcinus maenas*).

*Type location:* Mumbles Pier, Swansea Bay (51°34′8.958″N, 3°58′33.297″W).

*Type material:* Original slides used for this paper are stored together with biological material embedded in wax at Swansea University and the Cefas Weymouth Lab. The SSU rDNA sequence is deposited in GenBank under accession number MT311214.

*Etymology:* cranc (Welsh) = crab

## Discussion

Clear morphological differences were observed between the two new species, consistent with their disjunct branching positions in the phylogenetic analyses. *Haplosporidium* is well known to be a paraphyletic genus, and as more new sequence types are retrieved from infested animals and environmental sequencing studies, the more disunited lineages ascribable to the *Haplosporidium* become in the tree. It is clear that several new genera will be required once the necessary phenotypic data are available, and multi-gene trees will provide more insight into the branching order within the genus. Until such phylogenetic trees are available, attempts to determine the relationship between phylogeny and the subtle and multi-variate morphological differences between haplosporidian taxa are unlikely to be reliable, except at the highest level (for example between *Bonamia* and other haplosporidians).

Haplosporidian-like agents have been observed in crustaceans previously, e.g. in blue crabs *Callinectes sapidus* from North Carolina and Virginia, USA (Newman *et al*., 1976) and in a mud crab *Panopeus herbstii* from Grand isle, Louisiana, USA (Sprague, 1963), however, these studies do not benefit from the molecular phylogenetic approach that we have used in the current study for shore crabs. More recently, environmental sequencing studies and investigations of crustacean hosts have shown that many haplosporidian lineages are associated with crustaceans. Urrutia *et al*. (2019) described two new amphipod-infecting species of *Haplosporidium*, and revealed the diversity of crustacean-associated lineages across three diverse, but mostly poorly supported clades (consistent with the discussion above about the unsuitability of the 18S rRNA gene alone as a phylogenetic marker for haplosporidians). The two new species described here contribute further to our knowledge of crustacean parasites in this group. *Haplosporidium carcini* groups in Clade 1 of Urrutia *et al*. (2019), near the amphipod parasite *H. orchestiae* and three other crustacean-associated lineages, detected by haplosporidian-specific PCR of DNA extracted from crustacean tissues or sterile seawater in which putative hosts were incubated to investigate their epi/endobionts. The exception in that clade is *H. pinnae*, which is an emerging pathogen of the critically endangered pen shell or fan mussel (*Pinna nobilis*) in the Mediterranean (Catanese *et al*., 2018). The co-clustering of parasite lineages with a diverse range of host affiliations may turn out to be commonplace in haplosporidians, but it remains a valid hypothesis that the pen shell parasite may also be a parasite of crustaceans or has undergone a switch in host preference. The haplosporidian *Urosporidium crescens* is a hyperparasite of the encysted metacercaria of the digenean *Microphallus basodactylophallus* that infects the blue crab (*Callinectes sapidus*). This haplosporidian does not infect the crab tissue but rather it hyperparasitizes the trematode (Perkins, 1971). Although unidentified encysted trematode parasites were found in the hepatopancreas of four *H. carcini*-infected crabs, there was no evidence of any hyperparasitization.

*Haplosporidium cranc* groups in a clade of uncharacterized environmental sequences from coastal marine and brackish habitats in the UK and a marine fish farm in Borneo. The relationship of this clade to other haplosporidians is very unclear, with no strong affinities to any other clade in the group. On current phylogenetic evidence alone, it could easily be considered a separate genus, but more phenotypic data are required before such a decision could be made.

Although the prevalence of infection by both species of novel haplosporidians was low, the severity of infection was high in over 50% of parasitised crabs examined. For example, the haemolymph of most crabs was replete with uninucleate stages of both species of parasites and there was evidence of reduced numbers of haemocytes in circulation (as judged by their relative scarcity in haemolymph preparations) presumably as a result of infection. The milky, or opaque haemolymph observed although not a characteristic of haplosporidian infections alone, was also noted by Stentiford *et al*. (2013) in their study with *Haplosporidium littoralis* in *C. maenas*. Furthermore, cloudy haemolymph is found in *Hematodinium* infected crabs (Davies *et al*., 2019) and there is also a bacterial disease of shore crabs called milky disease, where the haemolymph is similarly opaque (Eddy *et al*., 2007). Hence, opaque haemolymph is not a unique characteristic of the current disease.

Despite the large numbers of parasites both in connective tissues around the hepatopancreatic tubules and in haemolymph, there was little clear evidence of any destruction or lysis of tissues as observed in other similar infections, including those caused by *Haplosporidium raabei* in zebra mussels *Dreissena polymorpha* (Molloy *et al*., 2012). Similarly, no stages of the parasites were observed infecting either free (i.e. circulating haemocytes) or fixed phagocytes in the gills and hepatopancreas unlike the observations of Stentiford *et al*. (2013) on *H. littoralis* infections in *C. maenas*. Deductions of intra- *vs* extra-cellular location of parasites can be difficult based on traditional histological methods alone and so we cannot fully rule out the possibility of intracellular development of these two novel haplosporidians. Furthermore, it is possible that these haplosporidians may replicate inside cell types in tissues not currently studied.

Many parasites and pathogens avoid or circumvent an immune response to aid in their invasion, therefore the lack of host response observed in our study was not an unusual finding. There are several examples of this phenomena in other infections within crustaceans. For instance, the dinoflagellate *Hematodinium* sp., a parasite of over 40 species of crustaceans, does not elicit any cellular host response (Rowley *et al*., 2015). Similarly, rickettsia-like bacteria also multiply in crabs inside fixed phagocytes in the hepatopancreas and hence can ‘hide’ away from the immune system (Eddy *et al*., 2007; Wang, 2011).

The histopathology of these two new species shows distinguishing morphological characteristics. In particular, the nuclei in uninucleate and plasmodia of *H. cranc* contain highly unusual chromatin bands seen both in live cells examined with phase-contrast microscopy and fixed, haematoxylin-stained cells in solid tissues while these are absent in both *H. carcini* (this study) and *H. littoralis* (Stentiford *et al*., 2013). Uninucleate forms in haemolymph are also less refractile than the equivalent stage in *H. carcini*. These features facilitate the rapid differentiation of these putative species based on morphology alone.

The presence of spore-like stages of both *H. carcini* and *H. cranc* was not observed in any of the tissues examined despite the acute nature of the infection as judged by the large numbers of parasites in the haemolymph. Similarly, Stentiford *et al*. (2004, 2013) noted this lack of an infective stage in *H. littoralis* infections in the same host species as the current study, and Urrutia *et al*. (2019) also found no evidence of sporulation in two new haplosporidian parasites of amphipods. Urrutia *et al*. (2019) suggested several reasons for this apparent lack of sporulating stages including the possibility that this event may occur in another host species. Shore crabs are scavengers and feed on a wide variety of dietary material such as dead fish and molluscs, including mussels (e.g. Cohen *et al*., 1995; Young and Elliott, 2020) leaving them vulnerable to chance infections. Indeed, the recent finding of low level infections of shore crabs by the important viral pathogen of molluscs, Ostreid herpesvirus-1 microVar (Bookelaar *et al*., 2018), may point to the possibility that *C. maenas* can act as a carrier, reservoir or alternate host for a range of parasites and pathogens because of their scavenging behaviour. The low prevalence of infections of *C. maenas* by these haplosporidians may also point to the presence of another definitive host in the intertidal zone where sporulation may occur.
